# Validating EEG source imaging using intracranial electrical stimulation

**DOI:** 10.1093/braincomms/fcad023

**Published:** 2023-02-07

**Authors:** Kanjana Unnwongse, Stefan Rampp, Tim Wehner, Annika Kowoll, Yaroslav Parpaley, Marec von Lehe, Benjamin Lanfer, Mateusz Rusiniak, Carsten Wolters, Jörg Wellmer

**Affiliations:** Ruhr-Epileptology, Department of Neurology, University Hospital Knappschaftskrankenhaus, Ruhr-University Bochum, 44892 Bochum, Germany; Department of Neurosurgery, University Hospital Erlangen, 91054 Erlangen, Germany; Department of Neurosurgery, University Hospital Halle (Saale), 06120 Halle, Germany; Ruhr-Epileptology, Department of Neurology, University Hospital Knappschaftskrankenhaus, Ruhr-University Bochum, 44892 Bochum, Germany; Ruhr-Epileptology, Department of Neurology, University Hospital Knappschaftskrankenhaus, Ruhr-University Bochum, 44892 Bochum, Germany; Department of Neurosurgery, University Hospital Knappschaftskrankenhaus, Ruhr-University, 44892 Bochum, Germany; Department of Neurosurgery, University Hospital Knappschaftskrankenhaus, Ruhr-University, 44892 Bochum, Germany; Ruhr-Epileptology, Department of Neurology, University Hospital Knappschaftskrankenhaus, Ruhr-University Bochum, 44892 Bochum, Germany; Besa, GmbH, 82166 Gräfeling, Germany; Institute for Biomagnetism und Biosignalanalysis, University of Münster, 48149 Münster, Germany; Otto Creutzfeldt Center for Cognitive and Behavioral Neuroscience, University of Münster, 48149 Münster, Germany; Ruhr-Epileptology, Department of Neurology, University Hospital Knappschaftskrankenhaus, Ruhr-University Bochum, 44892 Bochum, Germany

**Keywords:** source analysis, source reconstruction, source localization, inverse solution, head volume conductor modelling

## Abstract

Electrical source imaging is used in presurgical epilepsy evaluation and in cognitive neurosciences to localize neuronal sources of brain potentials recorded on EEG. This study evaluates the spatial accuracy of electrical source imaging for known sources, using electrical stimulation potentials recorded on simultaneous stereo-EEG and 37-electrode scalp EEG, and identifies factors determining the localization error. In 11 patients undergoing simultaneous stereo-EEG and 37-electrode scalp EEG recordings, sequential series of 99–110 biphasic pulses (2 ms pulse width) were applied by bipolar electrical stimulation on adjacent contacts of implanted stereo-EEG electrodes. The scalp EEG correlates of stimulation potentials were recorded with a sampling rate of 30 kHz. Electrical source imaging of averaged stimulation potentials was calculated utilizing a dipole source model of peak stimulation potentials based on individual four-compartment finite element method head models with various skull conductivities (range from 0.0413 to 0.001 S/m). Fitted dipoles with a goodness of fit of ≥80% were included in the analysis. The localization error was calculated using the Euclidean distance between the estimated dipoles and the centre point of adjacent stimulating contacts. A total of 3619 stimulation locations, respectively, dipole localizations, were included in the evaluation. Mean localization errors ranged from 10.3 to 26 mm, depending on source depth and selected skull conductivity. The mean localization error increased with an increase in source depth (*r*(3617) = [0.19], *P* = 0.000) and decreased with an increase in skull conductivity (*r*(3617) = [−0.26], *P* = 0.000). High skull conductivities (0.0413–0.0118 S/m) yielded significantly lower localization errors for all source depths. For superficial sources (<20 mm from the inner skull), all skull conductivities yielded insignificantly different localization errors. However, for deeper sources, in particular >40 mm, high skull conductivities of 0.0413 and 0.0206 S/m yielded significantly lower localization errors. In relation to stimulation locations, the majority of estimated dipoles moved outward-forward-downward to inward-forward-downward with a decrease in source depth and an increase in skull conductivity. Multivariate analysis revealed that an increase in source depth, number of skull holes and white matter volume, while a decrease in skull conductivity independently led to higher localization error. This evaluation of electrical source imaging accuracy using artificial patterns with a high signal-to-noise ratio supports its application in presurgical epilepsy evaluation and cognitive neurosciences. In our artificial potential model, optimizing the selected skull conductivity minimized the localization error. Future studies should examine if this accounts for true neural signals.

## Teaser

To evaluate the spatial accuracy of electrical source imaging (ESI) for known sources, Unnwongse *et al*. systematically performed ESI of averaged stimulation potentials recorded on simultaneous stereo-EEG and 37-electrode scalp EEG using individual finite element method models to determine the localization error.

## Graphical Abstract

**Graphical abstract:**
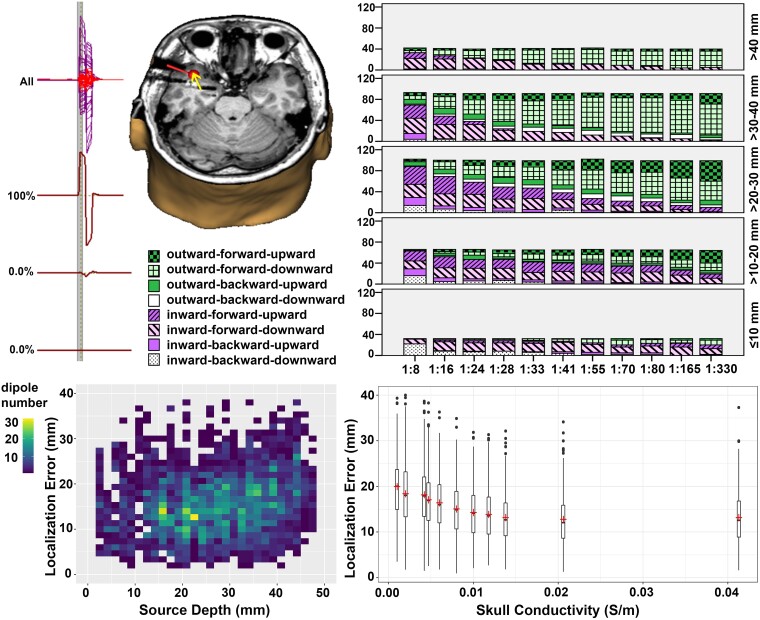

## Introduction

Electrical source imaging (ESI) of interictal and ictal epileptic activity has become an applied diagnostic tool in presurgical epilepsy evaluation.^1–5^ In epilepsy cases with concordant findings, ESI strengthens a hypothesis on the localization of the epileptogenic zone (focus hypothesis) which is based on seizure semiology, visual evaluation of interictal and ictal EEG, and MRI with epilepsy-specific sequences. In cases with discordant findings, possibly multifocal or non-lesional pharmaco-resistant epilepsies, ESI is applied to narrow the focus hypothesis.^6–8^ In particular, in non-lesional epilepsy, the result of interictal ESI serves to define targets of intracranial EEG electrode placement when the ictal onset on scalp EEG is not localizable or when the ictal onset is in the vicinity of the eloquent cortex.^9–11^ Technically comparable, the analysis of event-related potentials on EEG intends to localize neurophysiological processes in the brain.^12,13^ In either scenario, high spatial accuracy is essential. An ESI error of 2–3 cm might not affect decisions with regard to lobar resection, but is relevant for small resections, placement of intracranial electrodes or trajectories for radiofrequency or laser thermocoagulation.

A range of factors has been shown to influence the spatial accuracy of ESI. One of the most relevant is the number and coverage of EEG electrodes.^1,11,14^ Especially the use of additional infratemporal electrodes has demonstrated a positive effect on accuracy.^15^ The International Federation of Clinical Neurophysiology has recently published recommendations on a standard array including six electrodes of the inferior temporal chain, to capture epileptic activity from mesial temporal structures.^16,17^ However, numerous further important aspects of ESI have considerable impact, including applied head models,^18–21^ selected tissue conductivities,^22,23^ different inverse methods,^5,24^ spike selection and clustering,^25^ and time-point of ESI related to the spike (peak, half rising or spike onset).^26,27^ In particular, choosing optimal skull conductivity might be the most challenging, given that skull conductivity values are known to vary inter- and intra-individually and depending on measurement methods (*in vivo*, *ex vivo* or *in vitro*).^28,29^ Most available studies focus on diagnostic accuracy and clinical utility of ESI based on external validation. Namely, the concordance of ESI results with resection volume is evaluated in relation to postoperative seizure control.^9,10,30^ Since resection volumes may measure several centimetres in diameter, such studies, while clinically relevant, yield only coarse and indirect information on the spatial accuracy of ESI. Correspondingly, the comparison of ESI results with surgical resection volumes tends to overestimate specificity. Similarly, other studies report the overlap of ESI results with the focus hypothesis derived from other presurgical investigation techniques such as MRI (lesional and non-lesional), PET, single-photon emission computerized tomography, magnetic source imaging, EEG-functional MRI or electrocorticography, which also provide limited data on spatial accuracy.^2,10,31–34^

Results of studies validating ESI with consecutive intracranial EEG recordings may, however, suffer from limitations due to the variability of epileptic activity.^12,26,35,36^ It ultimately remains unclear whether ESI was calculated on patterns that are truly comparable to findings in the intracranial EEG, which may have been recorded weeks or months after the scalp EEG recordings. Ideally, ESI spatial accuracy should be evaluated using the same signal recorded simultaneously with both scalp and intracranial EEG.^37,38^ Probably due to the logistic effort required to combine scalp and intracranial EEG recordings, such studies are sparse. Comparison of ESI with stereo-EEG using such recordings found that the equivalent current dipole localization was on average 47.2 ± 23.2 mm away from the stereo-EEG seizure onset.^39^ While in another study using dipole and maximum distributed source, the median distance error to the centroid of seizure onset electrodes was 30–33 mm.^40^ However, validation of these studies is limited by subjective EEG seizure interpretation and restricted spatial sampling of stereo-EEG electrodes.^41^

The presented study, therefore, aims to evaluate the spatial accuracy of ESI using simultaneous stereo-EEG and 37-electrode scalp EEG during intracranial presurgical evaluation. To establish ground-truth localizations, we employed bipolar electrical stimulation of adjacent stereo-EEG contacts. ESI of the scalp EEG correlate of the stimulation potential was analysed and localization errors were calculated.

## Materials and methods

**Figure 1:**
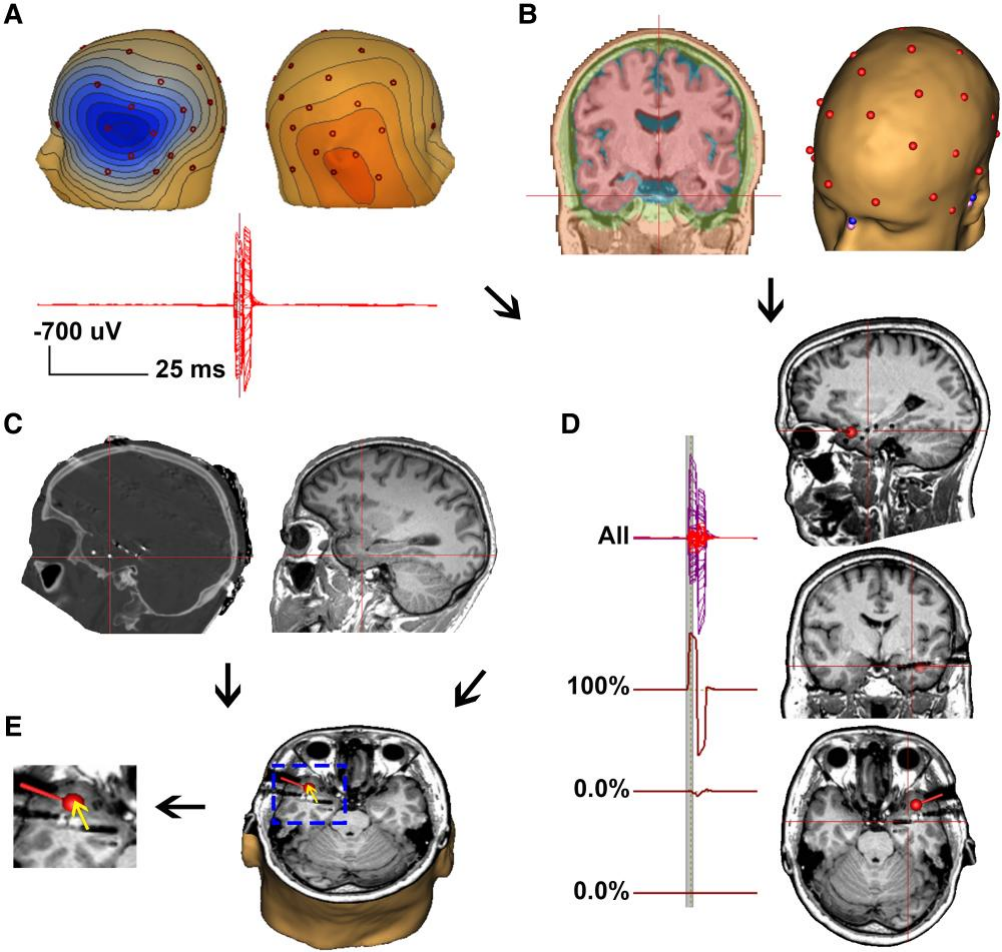
**Steps of the workflow.** (**A**) Scalp EEG channel over plot and topography map of the averaged stimulation potential was checked. (**B**) The individual pre-implantation MRI was segmented and scalp electrode data on the post-implantation CT were co-registered to the MRI to generate the individual FEM head model. Eleven skull conductivity values were applied to the individual FEM head model. (**C**) The locations of scalp and stereo-EEG contacts were identified on the post-implantation CT and the CT dataset was co-registered to pre-implantation MRI. Talairach coordinates of contacts were extracted from the MRI. (**D**) ESI of the averaged stimulation potential was performed using an individual FEM model. (**E**) Lastly, Talairach coordinates of stereo-EEG contacts from step (**C**) and Talairach coordinates of the estimated dipole from step (**D)** were used to analyse the localization error and the offset direction (arrow). Remark: post-implantation MRI in figure (**D**) and (**E**) are for illustration purposes.

Eleven patients who underwent intracranial stereo-EEG recordings as part of their presurgical evaluation for pharmaco-resistant focal epilepsy were studied. The decision to implant patients was made by a multidisciplinary team based on clinical parameters without consideration of the presented study. This study (register number: 20-6970) was approved by the ethics committee of the Faculty of Medicine, Ruhr-Universität Bochum, Germany. Simultaneous intracranial stereo-EEG and 37-electrode scalp EEG (10–20 system with additional paracentral and inferior frontal–temporal electrodes, Supplementary Fig. 1) were recorded for 1–3 weeks until a sufficient amount of interictal and ictal data were recorded. The number and placement of intracranial electrodes were determined according to the clinical context and the results of the previous non-invasive evaluations were not influenced by the goals of the presented study. Electrical stimulation was a part of our clinical evaluation during intracranial EEG recording.^42^ Stereo-EEG electrodes (AD-tech, Racine, WI, USA) with a diameter of 1.1 mm, contact length of 2.4 mm and inter-spacing length of 2.1 mm were used. Intracranial single-pulse electrostimulation (ISIS Stimulator, Inomed Medizintechnik GmbH, Emmendigen, Germany) of biphasic pulses (2 ms pulse width) at 1 Hz, 1 mA intensity, 99–110 trials, was applied on adjacent contact pairs in a bipolar fashion. Stimulation potentials on simultaneous stereo-EEG and 37-electrode scalp EEG were recorded on NeuroPort^TM^ (Blackrock Microsystems, UT, USA) with a sampling rate of 30 000 Hz. The steps of the workflow are shown in Fig. 1.

### Electrode localization

Isotropic T1 3D-magnetization-prepared rapid gradient-echo imaging volume MRI was acquired using a 3 T scanner (MAGNETOM Prisma 3.0T, Erlangen, Germany) before implantation of intracranial electrodes. Post-implantation CT (CT Elekta 1.0, Stockholm, Sweden) was acquired for localizing stereo-EEG and scalp EEG electrodes within 24 h after implantation. Electrodes were segmented using the post-implantation CT dataset. Individual stereo-EEG and scalp EEG contacts were labelled manually according to the clinical documentation. The CT dataset was then co-registered to pre-implantation MRI using a mutual information approach (Curry 7, Compumedics Neuroscan, USA, Fig. 1C).^43^ The resulting co-registered scalp electrode positions were used for ESI. The preoperative MRI was registered in Talairach space, which subsequently allowed expressing electrode localizations from CT in standardized Talairach coordinates and grouping according to anatomical regions. This whole process of electrode localization was done by one author (S.R.) in all patients. The locations of electrode contacts in different brain tissues (i.e. deep grey matter, white matter, grey-white matter junction and superficial grey matter) were visually identified by one author (K.U.) using post-implantation MRI.

### Electrical source imaging

Stimulation potentials were detected using a template search algorithm (BESA Research 6.1, BESA GmbH, Gräfeling, Germany). Templates, channels and settings for the automated search were selected manually. Detection results were checked for correctness and excessive additional artefacts. Scalp channels and a few trials with excessive noise, flat lines, etc. based on visual inspection were rejected (Fig. 1A). Stimulation potentials (99–110 trials) with an epoch length of −250 to 150 ms were then averaged. All filters (low and high pass as well as notch filters) were turned off, regularization was set to zero and baseline fit was set to −250 to −50 ms avoiding overlap with the stimulation pattern. A regional dipole was fitted from the onset to the negative peak with a fit interval of −1.5 to 0 ms of each averaged stimulation potential (Fig. 1D). ESI was done by one author, K.U. Fitted dipoles with a goodness of fit (GoF) of ≥80% were included in the analysis. An individual four-compartment finite element method (FEM) model with separate conductivity values of scalp (0.33 S/m), CSF (1.79 S/m), brain (0.33 S/m) and various skull conductivities was used (BESA MRI 2.0, BESA GmbH, Gräfeling, Germany). Values for skull conductivity were 0.0413, 0.0206, 0.0138, 0.0118, 0.01, 0.008, 0.006, 0.0047, 0.0042, 0.002 and 0.001 S/m, corresponding to a skull:scalp conductivity ratio (SSCR) of 1: 8, 16, 24, 28, 33, 41, 55, 70, 80, 165 and 330, respectively (Fig. 1B). We used SSCR instead of brain:skull conductivity ratio because brain compartment has the same conductivity value as scalp compartment and scalp and skull conductivity values are the most sensitive parameters on the EEG measurement.^21^ Each stimulation run (99–10 trials) using adjacent stereo-EEG contact pair thus yielded one averaged stimulation potential and subsequently one estimated dipole for each selected skull conductivity value. The coordinates of the estimated dipole and the centre point between adjacent stimulating contact pairs expressed as Talairach coordinates were determined. The Euclidean distance between these coordinates defined the localization error (Fig. 1E). Source depth was measured by the shortest distance from the inner skull (hull) to the centre of the stimulating contact pair. The offset direction was determined by the deviation of the estimated dipole from the centre of the stimulating contact pair along the horizontal, anterior–posterior and vertical axes of the head.

### Statistical analysis

Due to normally distributed data, mean and standard deviation (SD) were reported to present the centre and dispersion of the data. Relationship between the localization error and source depths as well as relationship between the localization error and skull conductivities were determined using curve estimation on SPSS 16.0 (IBM, New York, USA) to find a best-fit model. Pearson correlation was used to measure the strength of correlation between variables. One-way analysis of variance (ANOVA) and *post hoc* (Bonferroni) test were performed to compare the effect of selected skull conductivity values on mean localization errors at different source depths. Independent predicting factors of localization errors were identified using multiple linear regression (forward method).

## Results

**Figure 2:**
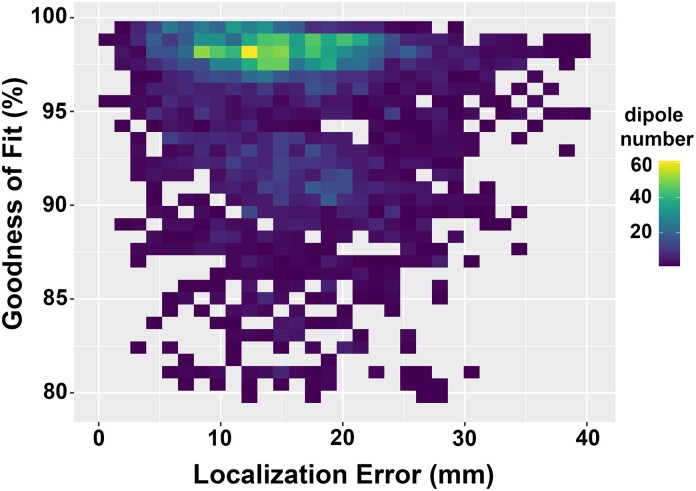
**Relationship between localization error and goodness of fit.** Density plot of all fitted dipoles with a goodness of fit ≥80% on localization error (*x*-axis) against goodness of fit (*y*-axis).

Eleven patients with pharmaco-resistant focal epilepsy undergoing intracranial evaluation with stereo-EEG electrodes implantation during 2016–18 were enrolled. A mean of six stereo-EEG electrodes (range 2–11) was implanted per patient with a mean of nine contacts (range 4–14) per electrode. In total, there were 620 contacts of 67 stereo-EEG electrodes. Fifty-five stereo-EEG electrodes were implanted in the temporal lobes and the remainder were located in the frontal lobes (Supplementary Fig. 2). Stimulation was applied across 553 contact pairs. EEG data from 212 contact pairs were excluded due to abundant artefacts (*n* = 108), amplitudes exceeding the amplifier’s dynamic range (*n* = 72) or incomplete stimulation trials (*n* = 32). Using the remaining contact pairs, 341 averaged stimulation potentials from 9 patients (2 women) were analysed with ESI, using 11 different skull conductivity values, resulting in 3751 estimated dipoles. Finally, 3619 (96.5%) fitted dipoles with a goodness of fit ≥80% were included in the analysis of localization errors (Fig. 2).

One hundred and thirty-two (3.5%) fitted dipoles showed a goodness of fit <80%. These fitted dipoles were derived, using 11 skull conductivity values, from ESI of 15 averaged stimulation potentials generated in 4 electrodes. Three of those electrodes belonged to one patient. One hundred and ten (83%) of these fitted dipoles had an average GoF of 75% (range 63–79), whereas the rest had an average GoF of 54% (range 50–60). Possible explanations of this low GoF are (i) technical issues such as contaminated data during stimulation or a defect of the particular electrodes as well as (ii) the distribution of scalp EEG electrodes of this particular patient did not cover both poles of the dipolar potential topography well enough.

### Localization error of estimated dipoles

**Figure 3:**
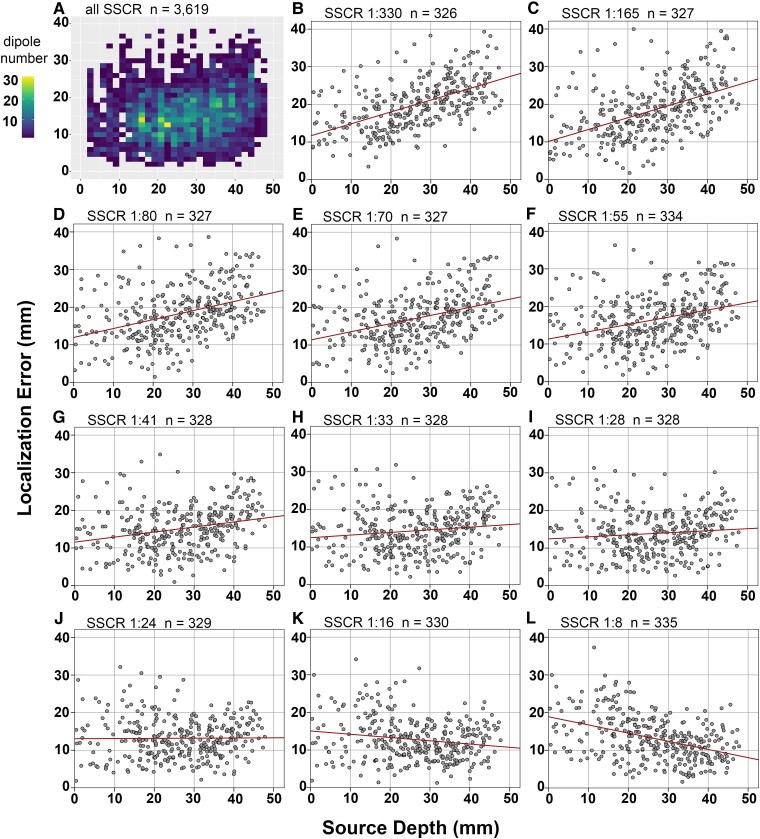
**Relationships between source depth and localization error.** (**A**) Density plot of dipoles on source depth (*x*-axis) against localization error (*y*-axis) across all SSCR. (**B–L**) Scatter plots of dipoles on source depth (*x*-axis) against localization errors (*y*-axis) in 11 SSCR (1:8 to 1:330, separate figures). (**B** and **C**) Based on the Pearson correlation, source depth and localization error were moderately correlated, *r*(324) = [0.55], *P* = 0.000 for SSCR 1:330 and *r*(325) = [0.49], *P* = 0.000 for SSCR 1:165. (**D**–**J**) For other SSCR, source depth and localization error were weakly correlated, *r*(325) = [0.38], *P* = 0.000 for SSCR 1:80 and 1:70, *r*(332) = [0.34], *P* = 0.000 for SSCR 1:55, *r*(326) = [0.26], *P* = 0.000 for SSCR 1:41, *r*(326) = [0.14], *P* = 0.01 for SSCR 1:33, *r*(326) = [0.11], *P* = 0.06 for SSCR 1:28, *r*(327) = [0.01], *P* = 0.86 for SSCR 1:24. (**K** and **L**) For SSCR 1:16 and 1:8, source depth and localization error were weakly inversely correlated, *r*(328) = [−0.18], *P* = 0.001 and *r*(333) = [−0.42], *P* = 0.000, respectively.

Plots of mean localization errors against source depths across various skull conductivity values showed linear relationships based on curve fit analysis [*R*^2^ = 0.04, *F*(1,3617) = 136.8, *P* = 0.000]. Overall, higher mean localization errors were observed with deeper source depth (*r*(3617) = [0.19], *P* = 0.000, Fig. 3). Moderate correlations of these two variables were found when using SSCR 1:165 and 1:330 (*r*(325) = [0.49], *P* = 0.000 and *r*(324) = [0.55], *P* = 0.000), and weak correlations were found when using SSCR 1:41 to 1:80 (*r*(325–332) = [0.26–0.38], *P* = 0.000). Using even higher skull conductivities (SSCR 1:8 and 1:16), the correlation of these two variables became inverse (*r*(333) = [−0.42], *P* = 0.000 and *r*(328) = [−0.18], *P* = 0.001), that is, higher mean localization errors were observed with more superficial source depth.

**Figure 4:**
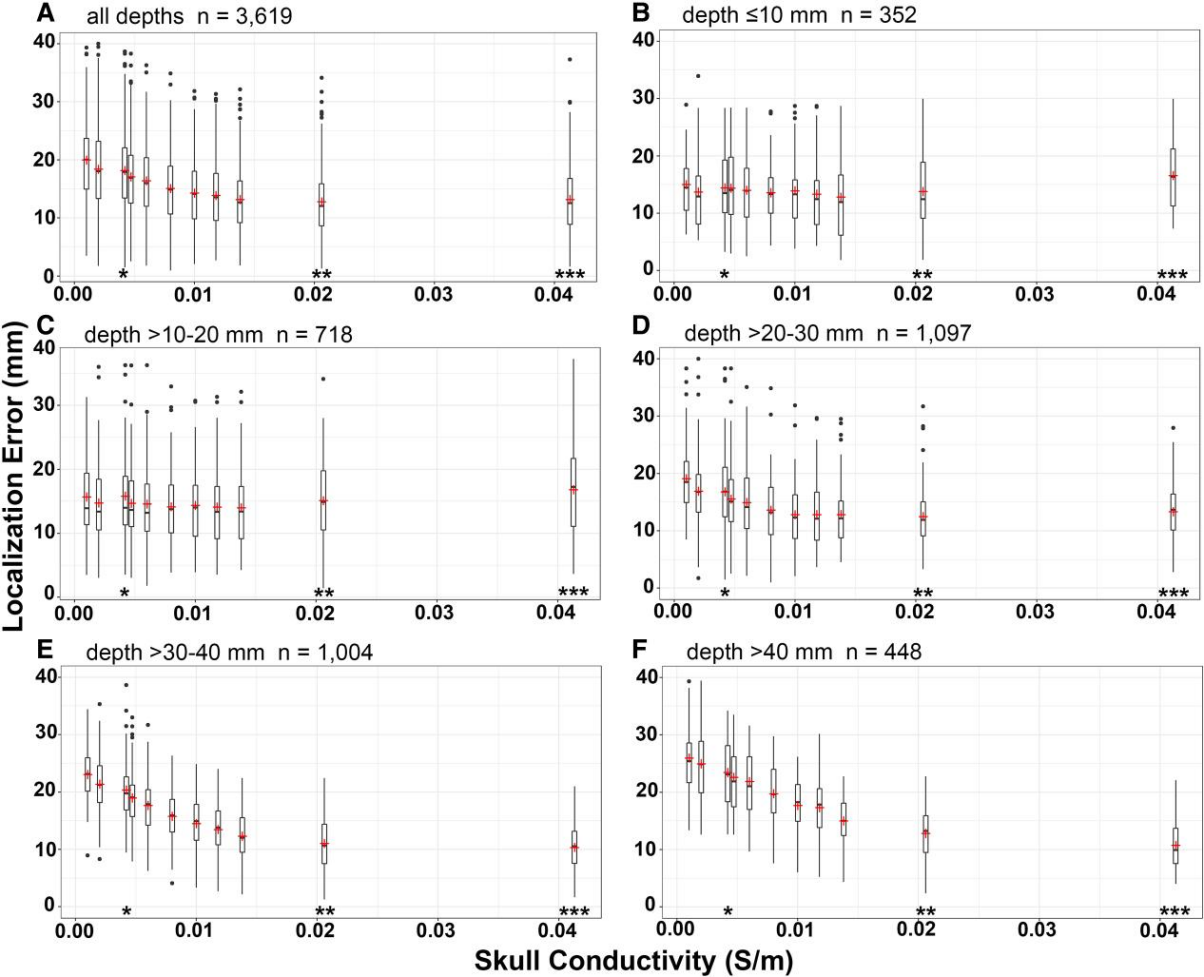
**Relationships between SSCR and localization error.** (**A**) Box plot of localization errors (*y*-axis) in relation to SSCR (*x*-axis) showing median localization errors and interquartile ranges across all source depths. (**B**–**F**) Box plots of localization errors in relation to SSCR in different source depth ranges. Crosses mark mean localization errors. *, **, *** are marked at SSCR 1:80, 1:16 and 1:8, respectively. (**B** and **C**) Based on the Pearson correlation, SSCR and localization error were not correlated, *r*(350) = [0.08], *P* = 0.16 for source depth ≤10 mm and *r*(716) = [0.06], *P* = 0.09 for source depth >10–20 mm. (**D**–**F**) For other depth ranges, SSCR and localization error were inverse correlated, *r*(1095) = [−0.2], *P* = 0.000 for source depth >20–30 mm, *r*(1002) = [−0.53], *P* = 0.000 for source depth >30–40 mm and *r*(446) = [−0.58], *P* = 0.000 for source depth >40 mm.

Plots of mean localization errors against skull conductivities across various depths mainly showed inverse linear relationships [*R*^2^ = 0.07, *F*(1,3617) = 251.1, *P* = 0.000], that is, higher mean localization errors were observed when using lower skull conductivity (*r*(3617) = [−0.26], *P* = 0.000, Fig. 4). Moderate correlations of these two variables were found at source depths of >30–40 and >40 mm (*r*(1002) = [−0.53], *P* = 0.000 and *r*(446) = [−0.58], *P* = 0.000), and weak correlations were found at source depths of >20–30 mm (*r*(1095) = [−0.2], *P* = 0.000). At more superficial source depths (≤20 mm), correlations were reverse to linear, that is, higher mean localization errors were associated with higher skull conductivities; however, these correlations were not statistically significant.

Using the standard adult skull conductivity (SSCR 1:80), mean localization errors ranged from 14.4 mm at a source depth of ≤10 mm to 23.4 mm at a source depth of >40 mm with SD of 7 and 6.4 mm, respectively (Table 1). At source depth ≤20 mm, one-way ANOVA revealed no significant differences in mean localization errors when using different skull conductivities from SSCR of 1:8 to 1:330. At source depth of >20 mm, one-way ANOVA revealed significantly lower mean localization errors when using higher skull conductivity values [>20–30 mm: *F*(10,1086) = 13.93, *P* < 0.01, >30–40 mm: *F*(10,993) = 71.48, *P* < 0.01, >40 mm: *F*(10,437) = 32.65, *P* < 0.01]. Multiple comparisons (Bonferroni’s test) revealed that at a source depth of >40 mm, SSCR of 1:8 yielded the lowest mean localization error, however, not significantly different from the error provided by SSCR of 1:16 (*P* = 1.0). At a source depth of >30–40 mm, SSCR of 1:8 yielded the lowest mean localization error, not significantly different from the errors provided by SSCR of 1:16 and 1:24 (*P* = 1.0). At source depth of >20–30 mm, SSCR of 1:16 yielded the lowest mean localization error, not significantly different from the errors provided by SSCR of 1:8 and 1:24 to 1:55 (*P* = 1.0). Across all depth ranges, the skull conductivity that yielded the lowest mean localization errors was SSCR of 1:16, however, not significantly different from the errors provided by SSCR of 1:8, 1:24 and 1:28 [one-way ANOVA: *F*(10,3068) = 50.89, *P* < 0.01 and Bonferroni’s test *P* = 1.0]. Using the standard adult skull conductivity, mean localization errors of 22.1 (SD ± 7), 18.1 (±6.5), 17.7 (±6.1) and 15.6 (±7.3) mm were observed with sources located in deep grey, white, grey-white and superficial grey tissues, respectively. The mean localization error with sources located in the mesial temporal structures (including the hippocampus, amygdala, parahippocampal gyrus and fusiform gyrus), lateral temporal cortex, lateral frontal cortex and mesial frontal cortex were 22.5 (SD ± 6.1), 14.4 (±5), 15.4 (±8.6) and 34.7 (±4.5) mm, respectively.

**Table 1:** Mean localization error and SD (mm), observed from sources located in different depths, tissues and anatomical regions, using 11 SSCR

SSCR	1:8	1:16	1:24	1:28	1:33	1:41	1:55	1:70	1:80	1:165	1:330
	*n* = 335	*n* = 330	*n* = 329	*n* = 328	*n* = 328	*n* = 328	*n* = 334	*n* = 327	*n* = 327	*n* = 327	*n* = 326
*Depth*											
>40 mm (*n* = 40–42)	10.7±4.4	12.8±4.6	15±4.4	17.3±5.4	17.7±5.1	19.7±5.5	21.9±6.1	22.6±6.2	23.4±6.4	25±7.1	26±5.9
>30–40 mm (*n* = 91–93)	10.3±4.2	11±4.2	12.3±4.4	13.5±4.8	14.5±5	15.8±4.9	17.7±5.2	19±5.2	20.4±5.5	21.4±5.7	23.1 ± 4.6
>20–30 mm (*n* = 99–102)	13.3±5	12.5±5.1	12.8±5.2	12.8±5.4	12.8±5.6	13.6±5.9	14.9±6.4	15.5±6.2	16.8±6.9	16.9±6.5	19.1±5.6
>10–20 mm (*n* = 64–66)	16.8±7	15.1±6.9	14±6.4	14.1±6.8	14.4±6.4	14.2±6.2	14.6±6.4	14.7±6.3	15.8±7.2	14.7±6.4	15.7±5.9
≤10 mm (*n* = 32)	16.6±6.2	13.8±6.9	12.8±6.9	13.3±7.1	13.9±7	13.6±6.2	14±7	14.4±6.9	14.4±7	13.7±7.3	15±5.7
All depths	13.2±5.9	12.8±5.6	13.2±5.4	13.9±5.9	14.3±5.8	15.1±6	16.4±6.6	17.1±6.6	18.2±7.1	18.4±7.3	20±6.5
*Tissue*											
Deep grey (*n* = 68–70)	11.5±4.8	13±5.1	14.6±5.2	16.2±6.1	16.9±5.9	18.7±6.3	20.6±6.6	21.4±6.8	22.1±7	23.5±7.4	24.6±5.7
White (*n* = 65)	12.4±6.4	12.3±6.1	12.7±5.3	13.8±6.3	13.7±5.8	14.4±5.5	16±5.8	16.8±6	18.1±6.5	18.9±6.2	21.1±5.4
White-grey (*n* = 92–93)	12.4 ± 6.4	12±5.1	12.6±4.8	12.9±4.5	13.4±4.8	14.2±5	15±5.3	16.3±6.4	17.7±6.1	17.7±5.7	19.3±5
Superficial grey (*n* = 90–92)	16.1±6	14.1±5.9	13.3±5.8	13.3±6.1	13.8±6.1	13.9±6.2	14.4±6.3	14.5±6.4	15.6±7.3	14.8±7.1	16±6.8
CSF (*n* = 11–15)	10.8±4.3	10±5.5	11.3±6.6	12.3±7.8	13.7±7.9	14±6.3	19.5±8.9	19.2±7.5	19.6±7.9	19.1±9.2	22.5±5.5
*Anatomy*											
Lateral temporal (*n* = 50–52)	16.7±5.8	14.6±4.8	13.5±4.4	13.3±4.6	13.6±4.6	13.5±4.4	13.5±4.6	13.4±4.5	14.4±5	13.4±4.2	14.9±4.8
Mesial temporal (*n* = 66–68)	11.2±4	12.9±4.3	14.7±4.4	16.4±5.5	17.1±5.2	18.8±5.5	20.9±5.6	21.8±5.9	22.5±6.1	24±6.6	25.1±5.3
Lateral frontal (*n* = 39)	15.3±6.5	12.7±7.6	12±7.4	12.2±7.6	12.8±7.5	13.3±7.7	14±7.6	14±7.2	15.4±8.6	14.5±8	15.5±6.7
Mesial frontal (*n* = 3)	21.5±3.9	22.6±4.9	25±4.9	25±4.9	27±5.6	29.5±5.8	30.9±4.6	33.3±4.6	34.7±4.5	36.8±3.1	36±2.2

### Offset direction of estimated dipoles

**Figure 5:**
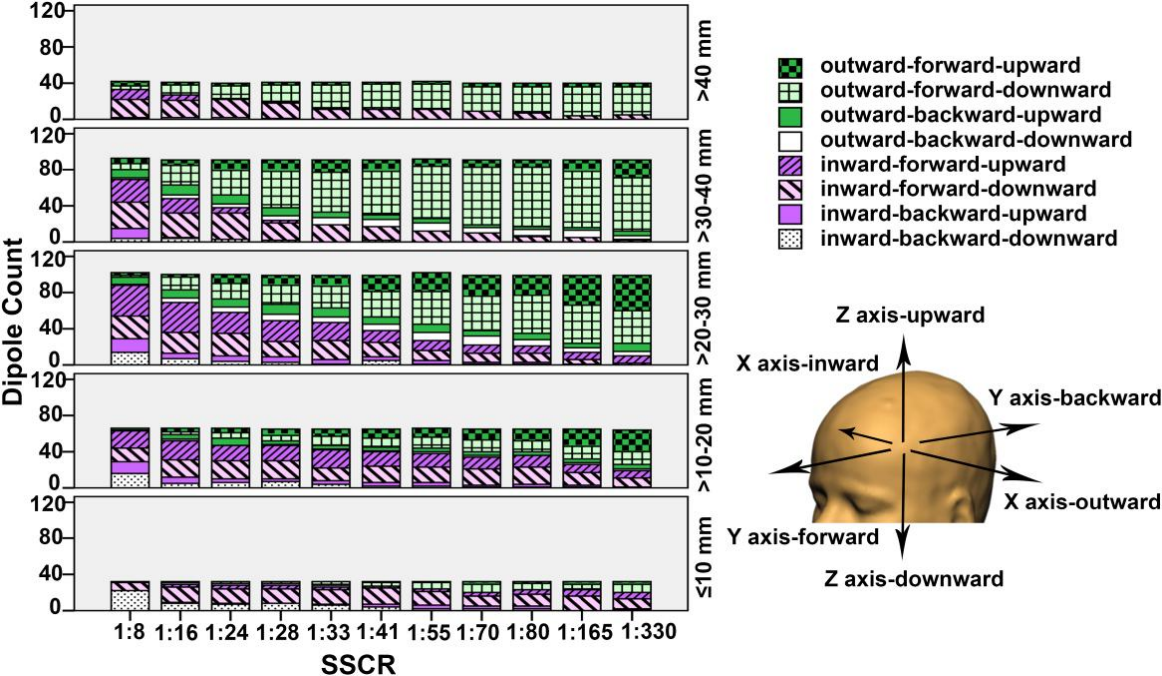
**The offset direction of estimated dipoles.** Diagram showing a number of dipoles (*y*-axis) on each category offset direction (block pattern) for all source depth ranges (each compartment) across all SSCR (*x*-axis).

The offset direction of estimated dipoles in relation to stimulation locations was evaluated in regard to the hemisphere, along the three axes of the Talairach coordinate system: horizontal in-out (*x*-axis), anterior–posterior (*y*-axis) and vertical (*z*-axis). On the horizontal axis, using a skull conductivity of 0.008 S/m (SSCR 1:41) and lower, 79, 90, 78, 51 and 29% of estimated dipoles, observed at source depths >40, >30–40, >20–30, >10–20 and ≤10 mm, respectively, moved outward from the centre of the head (Fig. 5). On the contrary, using SSCR higher than 1:41, 56, 43, 62, 77 and 89% of estimated dipoles, observed at source depths >40, >30–40, >20–30, >10–20 and ≤10 mm, respectively, moved inward to the centre of the head. On the anterior–posterior axis, the majority of estimated dipoles (80%) across various source depths and skull conductivities moved forward to the front of the head. Only a few estimated dipoles from source depths of ≤30 mm using high skull conductivities (SSCR 1:8 and 1:16) moved backward. On the vertical axis, across various skull conductivities, the majority of estimated dipoles (80%) observed at source depths >40, >30–40 and ≤10 mm moved downward towards the skull base. About 50% of estimated dipoles observed at remaining source depths moved downward. Overall, the offset direction mainly changed from outward-forward-downward to inward-forward-downward with a decrease in source depth and an increase in skull conductivity.

**Figure 6:**
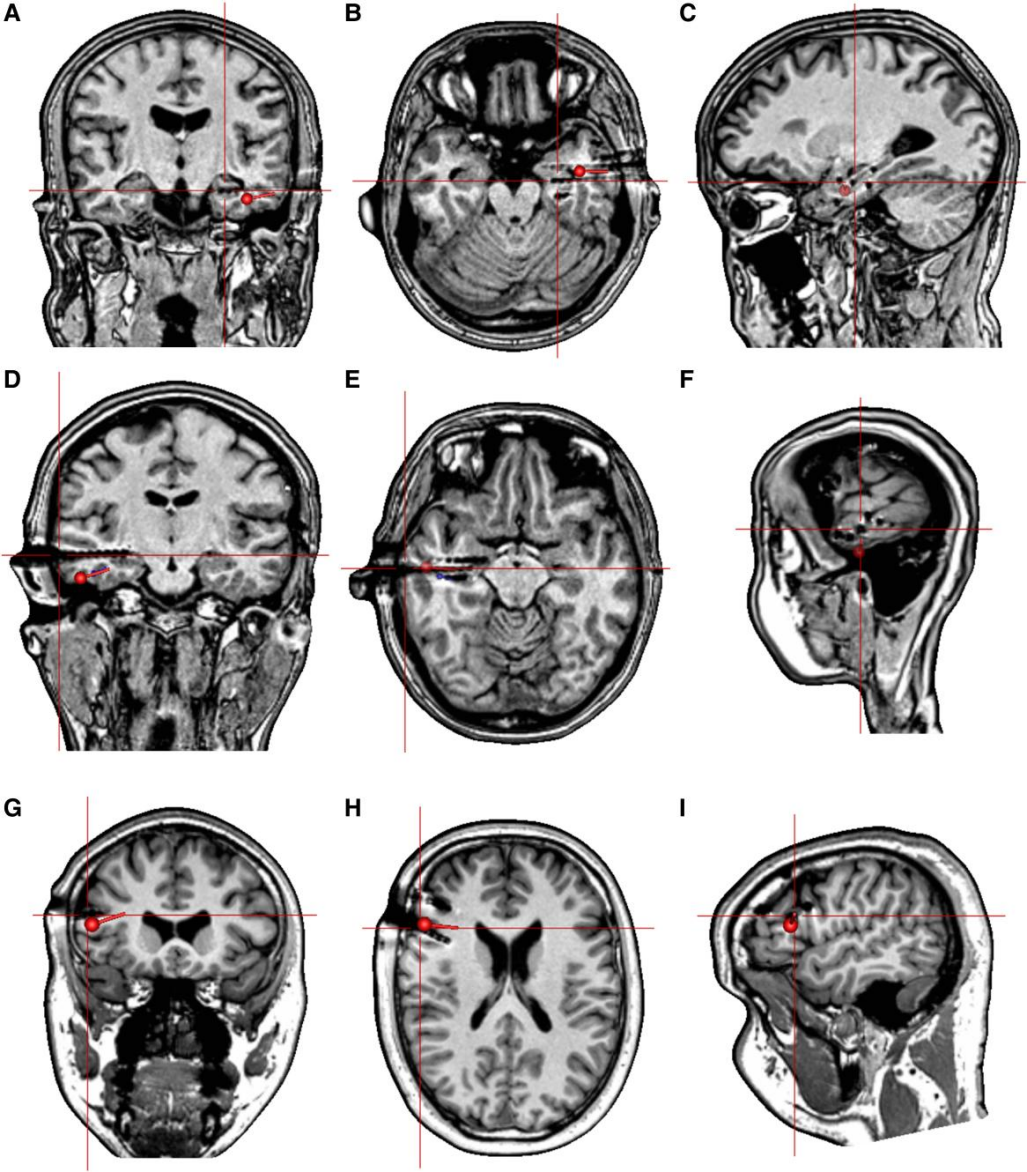
**The offset direction of estimated dipoles in different anatomical locations using standard adult skull conductivity (SSCR 1:80).** (**A**–**C**) The offset direction of the estimated dipole with the source located in the mesial temporal structure shifted outward-forward-downward, in relation to the location of stimulating contacts (crosshair). (**D**–**F**) The offset direction of the estimated dipole with the source located in the lateral temporal cortex shifted inward-forward-downward in relation to the location of stimulating contacts (crosshair). (**G**–**I**) The offset direction of the estimated dipole with the source located in the lateral frontal cortex shifted inward-forward-downward in relation to the location of stimulating contacts (crosshair). Remark: post-implantation MRI on the figures is for illustration purposes.

Using the standard adult skull conductivity (SSCR 1:80), the offset direction of estimated dipoles with sources located in mesial temporal structures (depth >40 mm) mainly shifted outward-forward-downward (67%) (Fig. 6). The offset direction of estimated dipoles with sources located in the lateral temporal cortex (depth >10–30 mm) shifted outward-forward-downward for 26%, outward-forward-upward for 22% and inward-forward-downward as well as inward-forward-upward for 18%. The offset direction of estimated dipoles with sources located in the lateral frontal cortex (depth ≤10 mm) shifted inward-forward-downward for 31% and outward-forward-downward for 21%.

### Further factors determining localization error

In multiple linear regression, the overall regression was statistically significant [*R*^2^ = 0.25, *F*(1,3609) = 23.4, *P* = 0.000], and four variables independently determining localization error were identified. Increase in source depth (*β* = 0.11; 95% confidence interval (CI)[0.09, 1.13]), number of skull burr holes related to the implantation of stereo-EEG electrodes (*β* = 0.89; 95% CI[0.79, 0.99]) and white matter volume (*β* = 0.04; 95% CI[0.03, 0.05]) as well as decreased skull conductivity (*β* = −154.4; 95% CI[−137.3, −171.5]) were associated with increased localization errors. This means that a 1 mm increment of source depth resulted in a localization error increase of 0.11 mm and with every 0.01 S/m decrement of skull conductivity, the localization error increased by 1.5 mm. One additional burr hole and 1 mm^3^ increment of white matter volume would increase localization error by 0.9 and 0.04 mm, respectively. Of the categorical variables, gender and anatomical region differences influenced localization errors. The two women had higher localization errors than men (*β* = 2.2; 95% CI[1.63, 2.76]). In comparison to sources located in the mesial temporal structures, sources located in the mesial frontal region had higher localization errors (*β* = 13.5; 95% CI[11.5, 15.5]), whereas sources located in the lateral temporal cortex (*β* = −1.49; 95% CI[−2.1, −0.89]) had lower localization errors. Grey matter and CSF volumes, skull thickness, number of stereo-EEG contacts and the subject’s age and whether stimulating contacts were located in grey or white matter did not show a systematic association with the localization error.

## Discussion

The present study evaluated the accuracy of ESI by measuring the localization error between the estimated dipole, analysed from the averaged stimulation potential, recorded on 37-electrode scalp EEG, and its known source, that is, the centre point between stimulating stereo-EEG contact pair. The main findings are that the mean localization error increased with an increase in source depth and a decrease in skull conductivity. Using standard adult skull conductivity (SSCR 1:80), the mean localization error of source depths 0.4–47.8 mm was 14.4–23.4 mm. Skull conductivity values of 0.0413–0.0118 S/m or SSCR 1:8 to 1:28 yielded significantly lower localization errors across all source depths. In relation to stimulation locations, the majority of estimated dipoles moved outward-forward-downward to inward-forward-downward with a decrease in source depth and an increase in skull conductivity.

### Localization error of estimated dipoles in relation to source depth and skull conductivity

The localization error associated with the source depth has been tested previously using electrical stimulation potentials or dipole simulation as the ground truth. In comparison to our study, these studies evaluated a smaller number of stimulation locations or simulated dipoles, a limited number of scalp EEG electrodes (21 electrodes, 10–20 EEG system), a spherical or boundary element method (BEM) head model, 1 selected skull conductivity value and different inverse solutions during ESI (Table 2). Cuffin *et al*.^44^ reported an average localization error of 11 mm (maximum 25.7 mm) based on analysis of 28 stimulation potentials with an unclear relationship between the localization error and the source depth. Krings *et al*.^47^ using 21 EEG electrodes found that the average localization errors of stimulating contacts located shallower in the temporal lobe (40–57 mm depth) were higher than errors of deeper contacts (62–85 mm depth). However, when adding 20 more electrodes on the anterior half of the head, according to the 10–10 EEG system, the average localization errors of deeper contacts were higher. The depth of sources in the Krings *et al*.^47^ study was much deeper than in our study. However, their average localization error observed using 41 scalp electrodes at source depth 40–57 mm was comparable to our average localization error at source depth >40 mm (19.3 versus 23.4 ± 6.4 mm, Table 1). In line with our findings, a dipole simulation study by Roth *et al*.^45^ showed that dipoles located in the mesial temporal area had higher localization errors than those located in the lateral and anterior temporal lobe or insula, with an average localization error of 19.7 mm (maximum 42 mm). Within the source depth range of 6–69 mm, Yvert *et al*.^46^ reported that the average localization error decreased with increasing source depth, discordant with our findings (Fig. 3). This probably resulted from numerical forward errors due to suboptimal mesh resolution. Our study used an appropriate mesh resolution; thus, numerical errors are assumedly negligible. With a higher number of simulated dipoles (*n* = 92), Whittingstall *et al*.^48^ found that the average localization error increased with an increase in source depth, similar to our findings, but the ranges of error were rather wide (∼30 mm). By means of electrical stimulation (61 locations), 256-electrode scalp EEG recording and realistically BEM as volume conductor model, Mikulan *et al*.^49^ showed that the choice of the inverse solution method employed during ESI affects the relationship between localization error and source depth. Using minimum norm estimate (MNE) and exact low-resolution electromagnetic tomography, the localization error increased with an increase in source depth with a strong correlation found for MNE (*β* = 0.7, *r*^2^ = 0.71). While using dynamic statistical parametric maps, the localization error increased with a decrease in source depth, with a weak correlation (*β* = −0.27, *r*^2^ = 0.27). Of note, the inverse solution is referred to a calculation of source characteristics from the measured (brain) potential distributions by considering the evolution of topographies over time (ESI), whereas the forward solution is referred to a calculation of a (brain) potential and its topography from a source with known characteristics.

**Table 2:** Studies evaluating the relationship between localization error and source depth

Study	Method	Brain area	Scalp EEG	Number of trials^a^	Head model	Skull conductivity (S/m)	Inverse solution	Average localization error (mm)	Source depth (mm)
Cuffin *et al*.^44^	Electrical stimulation	Frontal, temporal	21	12	Spherical	0.0042	Dipole	11	
Roth *et al*.^45^	Simulation	Frontal, temporal	21	8	BEM	0.0056		19.7	∼Mesial temporal
Yvert *et al*.^46^	Simulation	Parietal, temporal	21	24	Spherical, BEM	0.0056	Dipole	2–3	>30
								4–6	≤30
Krings *et al*.^47^	Electrical stimulation	Temporal	21	10	Spherical	0.0042	Dipole	19.3/16.4	40–57/62–85
			41					8.9/17	40–57/62–85
Whittingstall *et al*.^48^	Simulation	Parietal, temporal	21	92	BEM	0.0042	Dipole	35–65	35–70
								5–35	0–35
Mikulan *et al*.^49^	Electrical Stimulation	Frontal, Parietal, temporal	32–256	61	BEM	0.006	MNE	2–36	15–55
							dSPM	2–27	15–55
							eLORETA	2–21	15–55

dSPM, dynamic statistical parametric maps; eLORETA, exact low-resolution electromagnetic tomography.

a Number of stimulation locations or simulation dipoles.

In summary, the relationship between the localization error and the source depth observed from previous studies is likely influenced by factors including inverse solution methods, number of scalp EEG electrodes, depth of ground truth (source), number of measured stimulation locations or simulated dipoles, and accuracy of head modelling. Due to the simple and well-controlled source model as well as the well-validated dipole fit approach in our study, we are confident that the part of the error due to the inverse modelling is rather negligible. Errors are rather due to limited sensor number and coverage as well as limited forward modelling accuracy. To the best of our knowledge, this present study is the first to evaluate a large number of stimulation potentials of known dipolar sources (*n* = 3619) recorded simultaneously on stereo-EEG and scalp EEG, using dipole fit approaches in realistically shaped four-compartment FEM models. We found that an increase inf source depth independently led to higher localization errors.

Selection of skull conductivities plays an important part in solving the forward and inverse problem in ESI and significantly influences the localization accuracy in relation to source depth.^22,50–52^ Wang *et al*.^53^ found that if skull conductivities (SSCR 1:15 to 1:25) used for inverse solutions were higher than the conductivity (SSCR 1:80) used for the forward solution during the simulation, the localization error of deep sources (i.e. hippocampus) became lower. Conversely, if the skull conductivity used for the inverse solution was lower than the one used for the forward calculation, the localization error of superficial sources became lower. This is concordant with our finding that using an exceedingly high skull conductivity would result in a relatively low localization error for deep sources and a relatively high error for superficial sources (Fig. 4). Such over-estimation of skull conductivity moves the estimated dipole deeper into the brain.^54,55^ It is important to note that there is a methodological limit to this error, since the maximum depth in the source space is the centre of the head. This means that superficial sources have a larger ‘potential error range’ compared with deep sources. On the other hand, under-estimation of skull conductivity moves the estimated dipole closer to the inner skull. This effect is limited by the superficial boundary of the source space. Thus, deep sources, which have a larger potential error range, result in higher dipole localization errors. Currently, determination of individual skull conductivity is not feasible in a clinical setting. Based on a series of *in vivo* measurements, an SSCR of 1:15 may be more adequate than the standard SSCR of 1:80 due to its yield of higher accuracy.^56^ Conductivity values of the homogenized skull compartment are known to vary due to inter- and intra-subject variability and different measurement methods (*in vivo*, *ex vivo* or *in vitro*). In a meta-analysis of 20 studies, whole skull conductivity varied significantly, depending on the employed methodology (*P* = 0.02). Values for the whole skull conductivity obtained from electrical impedance tomography (∼0.006 S/m) were significantly lower than those obtained from directly applied current (∼0.0125 S/m) and electromagnetic data (E/MEG∼0.013 S/m). Based on 99 conductivity values from 121 participants in these 20 studies, a weighted average mean skull conductivity, calculated by taking into consideration the quality of each study, was 0.016 ± 0.019 S/m.^28^ This recommended value overlaps our range of skull conductivities (0.0413–0.0118 S/m; SSCR 1:8 to 1:28) that yielded significantly lower localization errors for all source depths. In particular, for sources deeper than 40 mm from the inner skull, SSCR 1:8 (0.0413 S/m) and 1:16 (0.0206 S/m) provided significantly lower localization errors compared with other conductivity values. However, due to inter-subject variability of skull conductivity and thickness, this common value may still result in considerable inaccuracies.^29^ If subject-specific calibrated realistic head models are not available, using a Bayesian uncertainty model with regard to unknown skull conductivity may result in improved localization.^29,55^

Evaluation of a broad range of skull conductivities in this study was influenced by our previous work.^50^ Following our experience on the difficulty of inter- and intra-subject variability in head tissue conductivities, using a scalp conductivity lower than 8 times skull conductivity (SSCR <1:8) is unrealistic for adult patients. Due to a gap in the range of evaluated SSCR between 1:16 and 1:8, we do not know if the localization error function is rather L-shaped or U-shaped. In the case of a U-shaped function, the minimum might be between the 1:16 and 1:8 ratios, while an L-shaped curve might be explained by constant localization errors as a result of using high skull conductivities for deep sources (Fig. 4). For deep sources (>40 mm), changes in skull conductivity influenced source magnitude rather than source localization.

### Offset direction of estimated dipoles

Previous studies reported the largest localization errors on the vertical axis with a marked downward shift of estimated dipoles.^45,47^ This may be caused by the limited electrode coverage of the inferior head by the 10–20 scalp EEG system. A whole head coverage, including face and neck, reduced the localization error for anterior temporal spikes because inferior head channels were important in measuring the ventral field distribution of the spikes.^15^ A hypothetical ‘full cap’ with evenly spaced electrodes around the entire head including the area below the skull base reduced localization errors in the centimetre range, compared with a realistic high-density EEG cap.^57^ Our mean localization error was the smallest 7.6 (SD ± 6.1) on the vertical axis, followed by 7.8 (±5.1) mm error on the anterior–posterior axis and 8.2 (±6.5) mm error on the horizontal axis. The additional inferior frontal–temporal coverage in our 37-electrode scalp EEG likely reduced the localization error on the vertical axis. A forward shift of estimated dipoles was observed using the 10–20 EEG system, but with an additional electrode according to the 10–10 EEG system on the anterior aspect of the head, which resulted in an opposite direction shift towards the back of the head, where the number of electrodes was lower.^47^ However, with our 37-electrode, 10–20 EEG system with additional paracentral and inferior fronto-temporal coverage, we again observed shifts in the forward direction of estimated dipoles. Our offset direction on the horizontal axis was mainly influenced by the selected skull conductivity applied during ESI.

### Further factors determining the localization error

We also found in a multivariate analysis that the localization error increased with increasing white matter volume; however, the effect size was small compared with the other factors discussed above. To the best of our knowledge, no studies have reported on the relationship between brain tissue volume and localization error. However, available evidence regarding the effects of tissue resistivity and anisotropy can at least in part explain our finding. Electric surface potentials (EEG) are sensitive to changes in the resistivity of the tissues located between the source and the scalp electrode.^58–60^ Tissue resistivity influenced ESI localization to a similar degree as anisotropy of grey and white matter.^61^ Sources placed in the sulcus next to white matter shifted >5 mm farther outwards and even more so when the source was surrounded by large white matter tracts, in particular, if anisotropic conductivity of white matter tissue was neglected in the FEM model.^62–64^ Inclusion of the grey/white matter distinction affected EEG magnitude and topography, which had implications for ESI localization.^19,21,65,66^ While using a realistically shaped four-compartment (scalp, skull, brain and CSF) FEM approach based on individual MRI, our head model still has limitations. Our individual FEM did not include scalp, grey and white matter conductivities that vary from one to another.^22,67^ Neither did we model other tissues such as dura and blood vessels.^68,69^ It is conceivable that the inclusion of these tissue types could create more accurate volume conduction models. Scalp conductivity uncertainties significantly influence EEG source localization, however to a lesser degree, compared with skull conductivity.^22^ Conductivity uncertainties of grey matter rather influence EEG forward solution than source localization, while white matter mainly affects the orientation of reconstructed sources.^22^ Further studies are required to clarify how the volume conduction parameters discussed above relate to ESI localization error.

Studies investigating the effect of skull defects on the localization error found errors of up to 10 mm when not modelling skull holes with a diameter of 5–20 mm in a volume conductor model using three-compartment (scalp, skull and brain) FEM models.^50,70,71^ Using five-compartment (scalp, compact bone, cancellous bone, brain and CSF) FEM models, if a skull hole was >6 mm in diameter and located in the proximity of the source, mean localization errors of only 1 mm were observed. A 2 mm diameter skull hole as used in our study resulted in negligible errors.^71^ However, we found that the localization error increased with the number of these ∼2 mm holes related to stereo-EEG electrode implantation, and the estimated impact was ∼0.9 mm per hole.

The influence of sex on localization accuracy was observed in our study. Women show a significant decrease in skull thickness (30–60%) with increasing age.^72^ Both an increase in age and a decrease in skull thickness are associated with a decrease in skull conductivity.^29,73^ The thickness of scalp layers varies with age and sex owing to hormonal differences.^74^ Local variations in skull and scalp thickness affect localization errors of about 1–6 mm.^71,75,76^ Based on these findings, it is conceivable that higher localization errors in women in our study may be due to differences in skull and scalp thickness. However, due to the low sample size of our cohort, a robust comparison was not possible.

Lastly, we found that sources located in the mesial temporal and frontal areas had higher localization errors than sources located in the lateral temporal and frontal cortices. Potentially due to the more sophisticated volume conductor model and the discrepancy of the anisotropic ratio in the mesial areas near the grey-white matter boundary together with a limited electrode coverage, large localization errors were reported for sources located mainly in the mesial and the basal aspects of the brain.^63^ Significant EEG magnitude and topography changes were found near interhemispheric and sylvian fissures if the larger CSF spaces in these areas were included in the head models.^19,21,77^ Moreover, sources in the basal aspect were susceptible to localization errors due to skull geometry, i.e. sinuses and thickness of the skull base if those were not modelled.^71^

### Limitations and future directions

We acknowledge the following limitations. First, our ESI results were based on 37 EEG electrodes placed according to the 10–20 system with additional paracentral and inferior frontal–temporal coverage. This comparably low number of electrodes may have diminished the accuracy of ESI in the range of a few millimetres to centimetres.^50,78,79^ In terms of diagnostic accuracy, a recent meta-analysis of both interictal and ictal source imaging for successful epilepsy surgery reported no significant differences in sensitivity, specificity and accuracy of ESI obtained from low (19–32) and high (65–256 electrodes) density EEG recordings.^3^ Simulation studies investigating spatial accuracy of ESI showed a significant influence of both the number of electrodes and their extended inferior coverage on the localization error.^15, 57^ Due to intrinsic limitations of volume conductor models and inverse solutions, localization errors were observed even when a high number of scalp electrodes were used in simulation studies and studies using electrical stimulation as known sources.^49,79^ In our experience, 37-electrode scalp video-EEG monitoring for presurgical evaluation is feasible over several days, is tolerable for patients and has limited additional technical requirements while increasing the yield for epileptic activity. With 37-electrode EEG, our mean localization error of ESI using standard adult skull conductivity was 14.4–23.4 mm from a source depth of 0.4–47.8 mm. This error compares favourably to the evaluation of lesions,^80^ resection^1,9,81^ and intracranial EEG.^28,82^ Nevertheless, further reducing this error must be the goal of future research. A further limitation is that the true skull conductivity values of individual patients were not available. Instead, we explored a range of skull conductivity values concerning the impact on ESI localization errors. Moreover, we did not vary tissue conductivity and include anisotropy of various compartments that influence localization accuracy in our head models, due to methodological and logistical limitations of our analysis pipeline and the applied software.^62,63,67,68,70,83,84^ The effect of exclusion of the stereo-EEG from our head models, however, might be negligible since our electric sources were Venant dipole sources around stereo-EEG contacts.^22,85,86^ Another limitation is that the stimulation potentials we examined probably have a much higher signal-to-noise ratio and do not necessarily exactly reflect the signal characteristics of true epileptic activity. Due to the attenuating property of the skull, the generating source of interictal epileptic activity is estimated to be at least 6–10 cm^2^ in order to be recognized on scalp EEG.^35,36^ For high-frequency oscillations (HFOs), one or a few small asynchronous oscillatory sources of about 1 cm^2^ contribute to scalp EEG.^87^ However, the signal-to-noise ratio of <1 in this case would make it difficult or even impossible to robustly detect such patterns. Thus, the accuracy of ESI of patterns with lower signal-to-noise ratios and less focal generators, such as HFOs and interictal epileptic activities, is likely to be lower compared with the artificial stimulation potentials used in our study. Our findings might thus overestimate the accuracy of ESI or at least represent a best-case scenario.

Moreover, there were intrinsic constraints of ESI that contribute to our remaining localization error. For once it is based on the quasi-static approach to Maxwell’s equations, ignoring capacitive, inductive as well as propagation effects that might contribute to our remaining localization error.^88,89^ Lastly, the FEM, implemented in BESA MRI, uses the Saint Venant source modelling approach. This method models a mathematical dipole source by means of a small cloud of monopolar sources in the close neighbourhood, which best approximates the intended dipolar moment. It assumes that the source is located in grey matter.^90^ In our study, this assumption holds when the two stimulating contacts are both in grey matter; however, if the stimulating contacts are in different tissues with different conductivities, the quality of the source model might suffer. In the future, a sensitivity study should be carried out to simulate these effects using, for example, a two-monopolar source.

Our findings of area-specific localization error magnitude and offset direction may be used to implement an error map that would allow annotating ESI results with information regarding the likely magnitude and direction of its mis-localization. Furthermore, individual skull conductivity could be calibrated using ESI of simultaneous intracranial and scalp EEG that would enable optimization of ESI results from previous scalp EEG recordings. Although such optimization would be late during presurgical evaluation, the findings might be informative for further electrode implantation or resection planning. However, such applications would have to be validated prospectively in a larger patient population considering different brain locations, a higher number of scalp EEG electrodes, more detailed head volume conductor models, etc.

## Supplementary Material

fcad023_Supplementary_Data

## Data Availability

The data that support the findings of this study are available from the corresponding author upon reasonable request.

## Abbreviations

*β* =: unstandardized coefficient
BEM =: boundary element method
dSPM =: dynamic statistical parametric maps
eLORETA =: exact low-resolution electromagnetic tomography
E/MEG =: electromagnetic data
ESI =: electrical source imaging
FEM =: finite element method
GoF =: goodness of fit
HFOs =: high-frequency oscillations
MNE =: minimum norm estimate
*r* =: correlation coefficient
SD =: standard deviation
SSCR =: skull:scalp conductivity ratio
