# Supplementary material for: Validating EEG source imaging using intracranial electrical stimulation
Source: Brain Commun. 2023 Feb 7;5(1):fcad023. doi: 10.1093/braincomms/fcad023 (PMC9942548; doi:10.1093/braincomms/fcad023)

Supplementary material

Supplementary Figure 1 37-scalp EEG electrode placement. Remark: FCz is ground and CPz is reference electrodes.

Supplementary Figure 2 a brain model showing 341 stimulation locations, located in temporal and frontal lobes.

Supplementary Figure 1

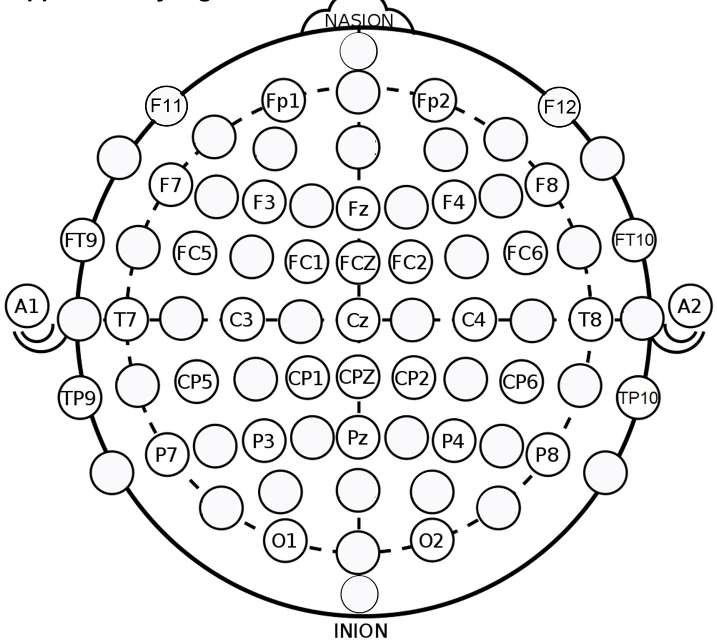

Supplementary Figure 2

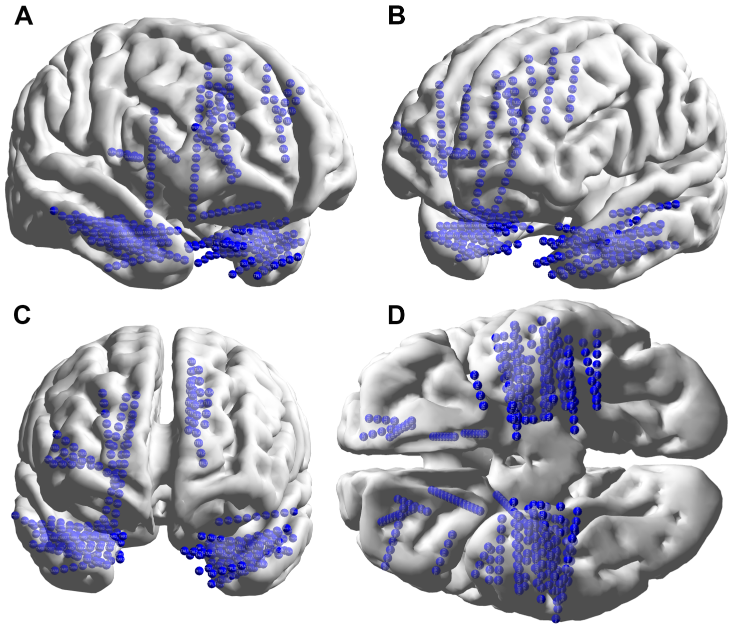

Supplement: fcad023_Supplementary_Data [file fcad023_supplementary_data.pdf]
